# Complex management of lentigo maligna in the setting of chrysiasis, argyriasis, and tattoo using in vivo reflectance confocal microscopy

**DOI:** 10.1111/1346-8138.16390

**Published:** 2022-05-05

**Authors:** Arthur Martin, Bruna Melhoranse Gouveia, Robert Rawson, Pascale Guitera

**Affiliations:** ^1^ The Melanoma Institute of Australia New South Wales Australia; ^2^ Medical School of The University of Sydney Sydney New South Wales Australia; ^3^ The Department of Tissue Pathology and Diagnostic Oncology Royal Prince Alfred Hospital Sydney New South Wales Australia; ^4^ The Sydney Melanoma Diagnostic Centre Royal Prince Alfred Hospital Sydney New South Wales Australia

**Keywords:** argyria, chrysiasis, confocal microscopy, hyperpigmentation, lentigo maligna

## Abstract

Lentigo maligna (LM) can be difficult to diagnose and recurrence is not uncommon. In vivo reflectance confocal microscopy (RCM) improves diagnostic accuracy of LM. LM can be difficult to discern from coexistent metal‐induced cutaneous hyperpigmentation (MICH). We are the first to describe three cases of LM associated with gold, silver, and metal oxide (from tattoos) and the RCM findings, respectively. The images obtained via RCM were analyzed by two RCM experts, and histopathology reviewed by a dermatopathologist. MICH under RCM appeared as intensely hyperreflective dots (when found freely) or clusters of variable sizes (when engulfed by macrophages) limited to the dermis. Dermal dendritic cells and melanophages were also found in association but distinct from the confluence of dendritic cells at the dermoepidermal junction observed in LM. We showed longitudinal changes within the dermis in MICH, not previously reported, where these hyperreflective dots congregate into clusters. RCM was able to distinguish the features of LM from MICH, delineate treatment margins, and monitor for recurrence.

## 1 INTRODUCTION

Lentigo maligna (LM) is a subtype of melanoma in situ that can be difficult to diagnose or delineate its extent.^1^ LM tends to occur on sun‐damaged skin of the head and neck region where surgery is cosmetically challenging. In vivo reflectance confocal microscopy (RCM) is a non‐invasive diagnostic tool with good histopathological correlation down to an upper dermal depth of 200 μm.^2^ Its diagnostic accuracy is superior to dermoscopy^2^ with 85% sensitivity and 76% specificity for diagnosing LM.^2^, ^3^ Metal‐induced cutaneous hyperpigmentation (MICH) can also be assessed under RCM.^4^, ^5^, ^6^, ^7^ However, use of RCM to differentiate features of LM from MICH has not been described previously.

## 2 METHODS

We explored the correlation of RCM findings and histopathology in a case series of three patients to show that RCM could differentiate the features of LM from coexisting dermal metal deposits in chrysiasis, argyriasis, and tattoo. Histopathology was reviewed by a dermatopathologist for correlation. All confocal images were analyzed by two confocal experts to establish agreement.

## 3 RESULTS

### 3.1 Case 1

**FIGURE 1:**
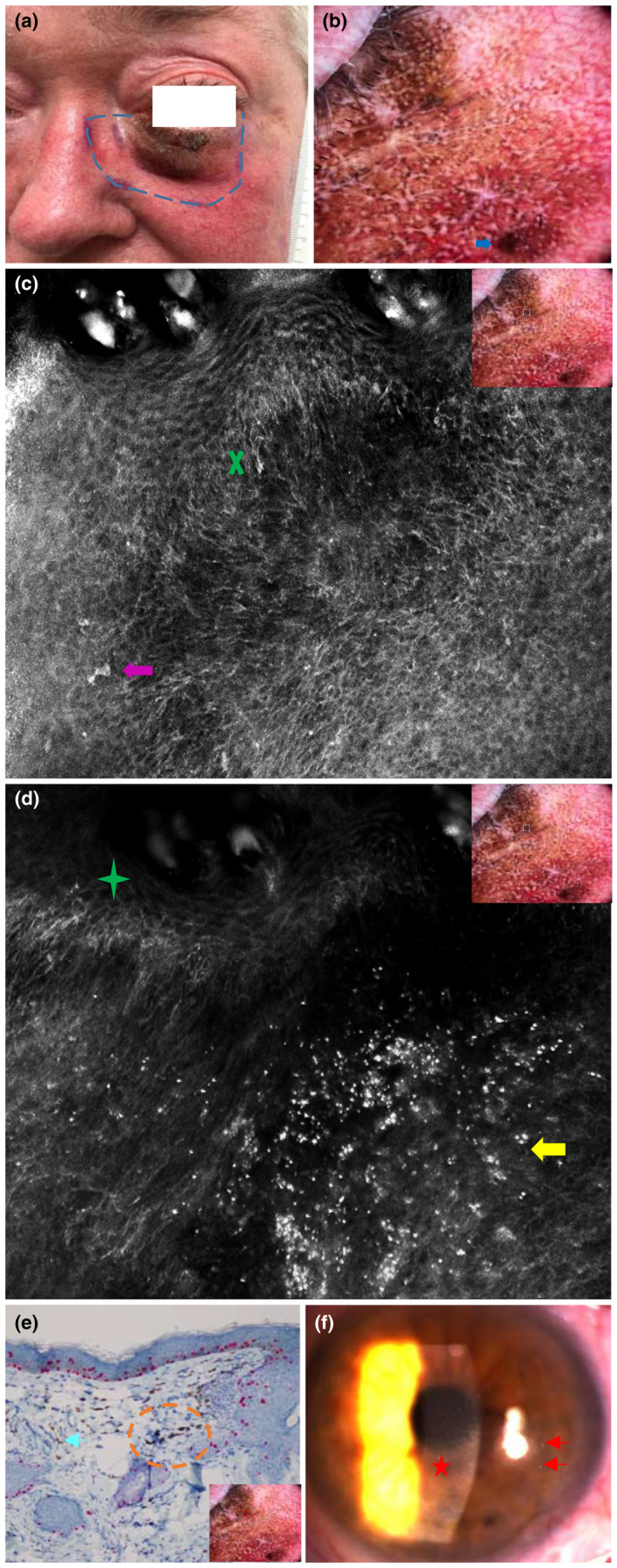
Lentigo maligna of the left lower eyelid on a background of generalized chrysiasis. (a) Clinical image of a dark patch on left lower eyelid. The purple dashes map the extent of disease observed under reflectance confocal microscopy (RCM). (b) Dermoscopic image, taken with DermLite 4 DL4 hand‐held dermoscopy (Macquarie Health), showing black dots of varying sizes arranged in an annular‐granular pattern forming asymmetrical pigmentation around follicular openings with some areas of obliteration of opening (thick blue arrow). (c) RCM imaging showing distortion at the dermoepidermal junction with confluent (green cross) dendritic cells and larger pleomorphic cells (thick purple arrow) that are highly indicative of lentigo maligna. Dermoscopic image indicates the location of RCM image taken (white dotted square). (d) RCM imaging displaying small and markedly hyperreflective dots and clusters in the dermis at the bottom right corner (thick yellow arrow) and dermoepidermal junction with distortion with small dendritic cells at the top left corner (green crosshair). Dermoscopic image indicates the location of RCM image taken (white dotted square). (e) Histopathology at ×200 magnification with SOX‐10 stain showing lentiginous pattern of mostly single melanoma cells along the dermoepidermal junction (staining red) and a few cells showing early upward spread. Gold deposits in extracellular matrix of the upper dermis appear as light brown globular structures (cyan arrow) and darker granules when engulfed by macrophages (orange dashes). Dermoscopic image indicates the location of biopsy taken (red circle). (f) Slit lamp examination of the anterior segment of the eye revealing fine gold particles (red arrows) and a few larger clusters in the cornea (red star)

A female in her 60s presented with a 4‐year history of pigmented patch on her left lower eyelid extending from the lash line, measuring 1.5 cm × 3.5 cm (Figure 1a). Dermoscopy showed broadened pseudo‐network with darkening, annular granular pattern, asymmetrical hyperpigmented follicular opening with signet‐ring circles predominantly, and obliteration of follicular opening (Figure 1b). An initial biopsy was reported as “post‐inflammatory pigmentation” but LM was confirmed upon biopsy 1 year later. She was also treated for rheumatoid arthritis associated with ulcerative keratitis.

Reflectance confocal microscopy imaging demonstrated pagetoid cells, epidermal disarray (with loss of regular honeycomb pattern), and confluence of atypical dendritic cells at the dermoepidermal junction (DEJ) which was indicative of LM (Figure 1c,d). There was no heterogenous melanoma nest or destruction of the DEJ to suggest dermal invasion. However, strikingly hyperreflective dots of irregular shapes and sizes were detected in the dermis (Figure 1e). It was revealed she had a decade‐long course of gold injections that stopped 5 years before. The contralateral cheek also displayed dermal hyperreflective dots. A lentiginous, junctional pattern of atypical cells staining positive for SOX‐10 (Figure 1e) on histopathology confirmed the diagnosis of LM. Additionally, dark brown/black globular structures found inside macrophages or within the extracellular matrix, which stained negatively for melanin, were thought to represent deposits of gold corresponding to the hyperreflective dots on RCM. These deposits were unlikely to be restricted to the skin. Her ophthalmologist confirmed corneal gold deposition (Figure 1f) that remained stable with no effect on vision. These substantiated the diagnosis of generalized chrysiasis. Treatment field was delineated via RCM (Figure 1a) by following the epidermal/junctional atypia. The patient received radiotherapy to a dose of 44 Gy in 22 fractions over 5 weeks. Post‐treatment monitoring with RCM over 2 years remained clear.

### 3.2 Case 2

**FIGURE 2:**
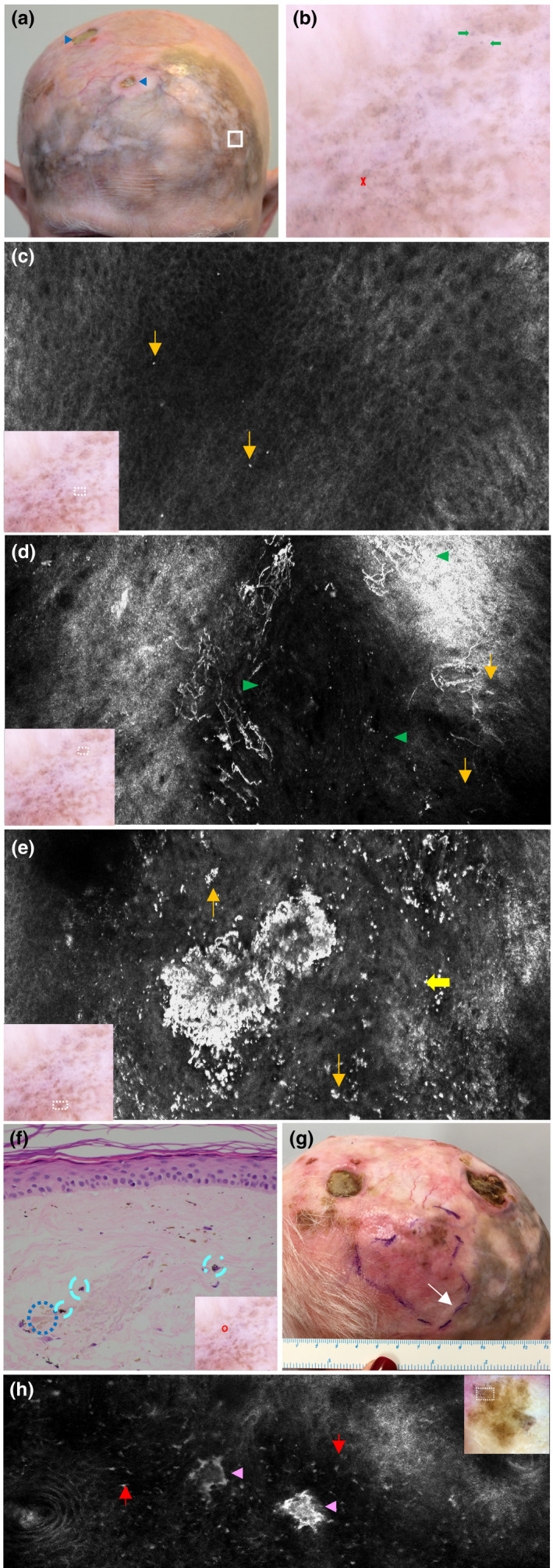
Previously treated lentigo maligna of the frontal scalp with localized argyriasis from silver dressings. (a) Clinical image of the extensive ill‐defined gray‐brown pigmentation interspersed with areas of depigmentation. There is a skin graft at the vertex where the desmoplastic melanoma was excised previously. There are also some chronic radiation‐induced necrotic ulcers (blue arrowheads). (b) Dermoscopic image (white square on image A) showing features of peppering (red cross), blue‐gray dots and globules (thick green arrows), and a few poorly‐defined tan blotches. (c) Reflectance confocal microscopy (RCM) imaging displaying an epidermis with regular honeycomb pattern (at the periphery) and normal dermoepidermal junction (center). A few small and scattered bright dots are observed within a small portion of the upper dermis in the center of the image (orange arrows). Dermoscopic image indicates the location of RCM image taken (white dotted square). (d) RCM imaging at upper dermis showing very bright dendritic cells (green arrowheads) and more small bright dots (orange arrows). Dermoscopic image indicates the location of RCM image taken (white dotted square). (e) RCM imaging at the upper dermis revealing a very bright star‐like cluster (thick yellow arrow) surrounded by many bright dots of varying sizes (orange arrows) consistent with the silver dermal deposits. These images of RCM were from the same stack through the layers of the skin. Dermoscopic image indicates the location of RCM image taken (white dotted square). (f) Histopathology at ×400 magnification with hematoxylin–eosin stain of skin with no significant melanocytic proliferation. Within the superficial dermis, there are free silver deposits in the extracellular matrix seen as faint‐brown globules (blue dotted circle) and macrophages with silver‐containing granules (cyan dashes). Severe elastosis is also noted. Dermoscopic image indicates the location of biopsy taken (red circle). (g) Clinical image of RCM mapping of lentigo maligna (LM) occurring in right parietal scalp posterior to the previous radiotherapy field, delineated by the purple dashes; the 3 o’clock margin is at the edge of the localized argyriasis (white arrow). Surgical margin had included 5 mm beyond the mapped area and the histopathological report showed that the 3 o’clock margin was clear of disease by 8 mm. (h) RCM imaging of the amelanotic area within the delineated recurrence of LM displayed heterogenous nests (pink arrowhead) among a confluence of comma‐like atypical cells (red arrow) in a hyporeflective background. Dermoscopic image indicates the location of RCM image taken (white dotted square)

A male in his 70s had a complex history of desmoplastic melanoma with Breslow thickness of 4 mm on his scalp that was initially excised 12 years before. Recurrence as LM over a large area across the frontal scalp was detected 6 years before and treated with radiotherapy (56 Gy in 26 fractions). The irradiated area developed wet desquamation treated with 1% silver sulfadiazine cream. The patient consequently self‐medicated with 25 tubes (50 g) of the latter over several months. An irregular area of gray pigmentation persisted on his anterior scalp and forehead (Figure 2a) with dermoscopy features of gray dots and globules interspersed with tan blotches but no features to suggest LM (Figure 2b). RCM imaging identified hyperreflective dots in the dermis with regular, honeycombed epidermis, and intact DEJ (Figure 2c) without features of LM.

Six‐monthly monitoring with RCM revealed long, bright dendritic cells interspersed with hyperreflective dots throughout the upper dermis but none at DEJ (Figure 2d). These clearly hyperreflective dots had progressively aggregated to form large clusters which did not resemble nests of melanoma cells. These intensely hyperreflective clusters had no cellular component but rather sharp demarcated structures (Figure 2e). LM was excluded on biopsy but brown pigmentation and clusters of darker globules were found within the dermis. These were interpreted as silver deposits found freely or packed in granules within melanophages (Figure 2f). Surveillance via RCM 5 years later detected recurrence of LM on the right parietal scalp (Figure 2g). Heterogenous nests of melanoma within a hyporeflective, structureless surrounding were identified under RCM in the amelanotic area of suspected recurrence (Figure 2h). Surgery was undertaken with margins guided by pre‐operative RCM (Figure 2g). The anterior margin, adjacent to the localized argyriasis, was clear of LM cells by 8 mm. RCM was able to exclude LM despite the silver deposits. He died a year later of complications from non‐related comorbidities.

### 3.3 Case 3

**FIGURE 3:**
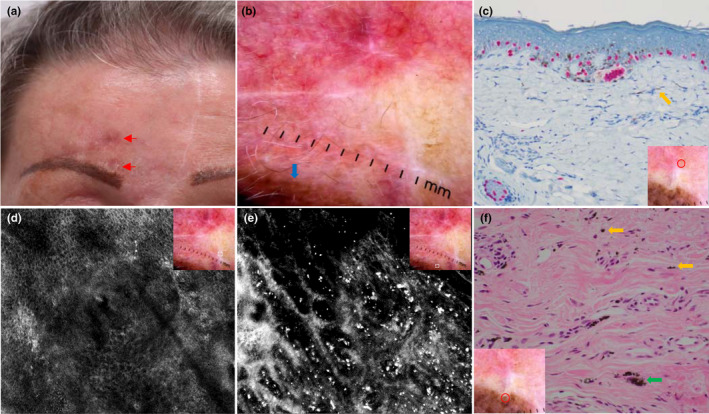
Recurrent lentigo maligna (LM) adjacent to an eyebrow tattoo. (a) Clinical image of a brown macule with recent biopsy showing early LM (red arrows) and a long vertical scar above the glabella from the multi‐staged excision of previous LM. Note the eyebrow tattoo inferior to the suspicious site. (b) Dermoscopic image displaying tan brown blotches at the biopsy scar and dark brown pigmentation of the eyebrow tattoo (thick blue arrow); there are no obvious features of LM. (c) Histopathology at ×200 magnification with SOX‐10 stain displaying increased crowded, single and focally confluent melanocytes with mild atypia suggestive of early focal transition to LM. There are small amounts of pigmentation in the extracellular matrix of the dermis (thick orange arrow). Dermoscopic image indicates the location of biopsy taken (red circle). (d) Reflectance confocal microscopy (RCM) imaging showing normal dermoepidermal junction at the center of the image and pigmented keratinocytes within the epidermis of mildly atypical, less well‐defined honeycomb pattern. Dermoscopic image indicates the location of RCM image taken (white dotted square). (e) RCM imaging demonstrating multiple bright dots throughout the papillary dermis. Dermoscopic image indicates the location of RCM image taken (white dotted square). (f) Histopathology at ×400 magnification with hematoxylin–eosin stain of the dermis with free tattoo metal oxides in the extracellular matrix (thick orange arrows) aggregating to larger clusters (thick green arrow). Dermoscopic image indicates the location of biopsy taken (red circle)

A female in her 70s had an extensive amelanotic LM on the glabella 5 years before and required 3 successive excisions to achieve good clearance. One year prior, a 1 cm × 1.5 cm pink‐brown macule developed above her right eyebrow (Figure 3a) with bland dermoscopy features of tan blotches (Figure 3b). Biopsy showed lentiginous proliferation of atypical melanocytes which stained positive on SOX‐10 and S100 stains (Figure 3c), suggestive of early LM. She was referred for RCM mapping to define the lesion margins. Notably, her eyebrows were tattooed previously. We observed features of sun damage under RCM but none to suggest LM (Figure 3d). There were intensely hyperreflective dots with no cellular component mostly within the confines of the tattoo (Figure 3e). It was favored that they represented metal oxide deposits commonly used in tattoo inks (Figure 3f). Surveillance with RCM had not shown any recurrence for the past 2 years.

## 4 DISCUSSION

To the best of our knowledge, these three cases are the first reports on coexistent MICH and LM assessed with RCM. We also describe the first cases of exogenous dermal deposits from generalized chrysiasis (case 1) and tattoo (case 3) examined under RCM.

Previous reports of RCM studies detecting chrysiasis and argyriasis had similarly observed hyperreflective structures in the upper dermis.^4^, ^5^, ^6^, ^7^ Fuchs et al. (2020) described a case of a 17‐year‐old female with localized chrysiasis from therapy combining topical gold microparticles followed by laser therapy to treat acne vulgaris. RCM imaging of a blue‐gray macule that developed during treatment displayed hyperreflective pinpoint structures and clusters. They suggested that the latter were aggregates of macrophages dispersed in the papillary dermis. An 8‐month follow‐up with RCM showed that the macrophages had disappeared and only pinpoint structures remained.^4^ However, in our case 2 of localized argyriasis, progressive aggregation of macrophages as large clusters were detected on 6‐year RCM monitoring. These findings may be due to a larger area exposed to silver at much higher doses.

Atypia in MICH is only observed in the dermis under RCM. These aggregates of macrophages with engulfed deposits are not to be confused with melanoma cells which are observed as dendritic or round cells with pleomorphism and/or nucleation under RCM.^3^ From our observation, metal deposits engulfed by macrophages are more intensely hyperreflective compared to melanin in melanoma cells.

Under RCM, melanoma cells can aggregate as nests seen along DEJ. Melanoma nests have been described as polyhedral (dense cluster) or roundish non‐reflective/inhomogeneous structures (sparse nest),^8^ and tend to be smaller in LM. Melanoma nests are distinguishably less reflective or defined than the star‐like clusters of macrophages in metal‐induced hyperpigmentation. LM can also be associated with melanophages which are triangular‐shaped and smaller than melanoma cells under RCM.^9^ Melanophages are less reflective than macrophages containing engulfed metal compound. Additionally, we found long dermal dendritic cells of localized cutaneous argyriasis. These are Langerhans cells involved during an inflammatory response. These cells have been described under RCM during and, reportedly, persisting up to 42 months post‐radiotherapy for the treatment of LM (Table 1).^10^, ^11^

**TABLE 1:** Features of lentigo maligna (LM) and metal‐induced cutaneous pigmentation under in vivo reflectance confocal microscopy (RCM)

	Lentigo maligna		Metal‐induced cutaneous pigmentation	
Clinical manifestation	Atypia only found in area of disease		Can be found on skin of any part of body when there is generalized deposition (case 1), or focal area of skin when there is localized deposition of metal compounds (case 2 and 3)	
Topography on RCM	Atypical features observed in epidermis (pagetoid cells), dermoepidermal junction (distortion with confluent dendritic or round cells) and dermis (elastosis; melanophages)^3^		Metal dermal deposits located extensively throughout and limited to the dermal layer of the skin	
Morphology on RCM	Melanoma cells are dendritic or round in shape, display pleomorphism, and can be nucleated	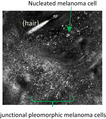	Metal dermal deposits (either found freely in the dermal extracellular matrix or engulfed by macrophages) appear as brilliantly and homogenously hyperreflective small dots of varying sizes	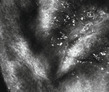
	Nest: Nest of melanoma cells are polyhedral/dense or roundish/inhomogenous	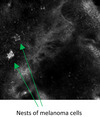	Cluster: macrophages containing metal deposits aggregate together, appearing as larger hyperreflective structures with well‐defined outlines (refer to case 2). Clusters can also be a result of direct injection of large amount of metal deposits into the dermis in the case of tattoos (refer to case 3)	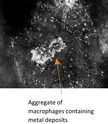
	Melanophages: LM can be associated with melanophages, that usually are not in clusters. They are in triangular‐shaped and smaller than melanoma cells The upper dermis in LM can also have small inflammatory cells These are in keeping with pigment incontinence	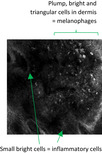	Macrophages: in the upper dermis, macrophages containing engulfed. foreign metal deposits can aggregate in varying numbers, thus appearing as smaller, well‐defined, hyperreflective clusters	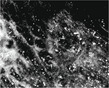
	Dermal dendritic cells are frequently observed during radiotherapy treatment of LM	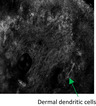	Dermal dendritic cells of increased numbers are sometimes seen as part of inflammatory response when large amounts of dermal foreign deposits are introduced locally (refer to case 2)	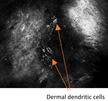

Reflectance confocal microscopy identified the extent of atypia within the epidermis and DEJ for the diagnosis and treatment of LM, and exclude dermal invasion despite dermal metal deposits. RCM assisted in achieving good clearance of surgical margins (case 2), guided the radiotherapy field (case 1) and avoided extensive excision when monitoring was preferred (case 3).

In conclusion, RCM is an ancillary to the diagnostic process of LM. It is useful in differentiating dermal deposits from native cutaneous structures or atypical cells of LM. RCM can also be used in the follow‐up of LM and its differentials, including MICH.

## CONFLICT OF INTEREST

None declared.
